# 3D Printing of Continuous Fiber Reinforced Low Melting Point Alloy Matrix Composites: Mechanical Properties and Microstructures

**DOI:** 10.3390/ma13163463

**Published:** 2020-08-06

**Authors:** Xin Wang, Xiaoyong Tian, Lixian Yin, Dichen Li

**Affiliations:** 1 State Key Laboratory of Manufacturing Systems Engineering, Xi’an Jiaotong University, Xi’an 710049, China; wx369806199@hotmail.com (X.W.); yinlixian@stu.xjtu.edu.cn (L.Y.); xjtudcli@sina.com (D.L.); 2 Shaanxi Provincial Rapid Manufacturing Engineering Technology Research Center, Xi’an 710049, China

**Keywords:** 3D printing, wicking performances, metal matrix composites, interfaces

## Abstract

A novel 3D printing route to fabricate continuous fiber reinforced metal matrix composite (CFRMMC) is proposed in this paper. It is distinguished from the 3D printing process of polymer matrix composite that utilizes the pressure inside the nozzle to combine the matrix with the fiber. This process combines the metallic matrix with the continuous fiber by utilizing the wetting and wicking performances of raw materials to form the compact internal structures and proper fiber-matrix interfaces. CF/Pb50Sn50 composites were printed with the Pb50Sn50 alloy wire and modified continuous carbon fiber. The mechanical properties of the composite specimens were studied, and the ultimate tensile strength reached 236.7 MPa, which was 7.1 times that of Pb50Sn50 alloy. The fracture and interfacial microstructure were investigated and analyzed. The relationships between mechanical properties and interfacial reactions were discussed. With the optimized process parameters, several composites parts were printed to demonstrate the advantages of low cost, short fabrication period and flexibility in fabrication of complex structures.

## 1. Introduction

Metal matrix composite (MMC), especially the continuous fiber reinforced metal matrix composite (CFRMMC), which has the advantages of high specific strength, high specific modulus, low density, corrosion resistance and oxidation resistance, is a revolutionary, futuristic type of metal [1,2,3,4]. Lu [1] indicates MMC would be an alternative material to aluminum and titanium alloys, and the development of MMC is necessary.

In recent years, composites, especially continuous fiber reinforced composites, fabricated by 3D printing [5,6,7] have become one of the research tendencies, and the developments are rapidly. Tian [8] studied the 3D printing of continuous carbon fiber reinforced PLA composites and realized the rapid fabrication of complex composite structures with low cost. Hou [9] printed the corrugated structure composites with aramid-fibers/PLA, and the maximum compression strength reached 17.17 MPa. Parandoush [7] showed the 3D printing of composites had the advantages of process flexibility and high-performance. Recent research demonstrated the feasibilities of 3D printing to fabricate high performance and complex structure composites with low cost by the fused-extrusion process. Now, the printable matrix materials are limited in resin, which are prone to failure under the extreme conditions, such as high temperature and radiation. The studies of metal matrix composite fabricated by 3D printing are necessary.

Comparing with the polymers, Mills [10] indicated the surface tension of molten metal was considerably higher and melt viscosity was much lower. In conventional fabrication process of CFRMMCs, high pressure differential was always necessary in order to achieve less internal defects and good interfaces [11,12,13,14]. Shirvanimoghaddam [15] indicated the pressure could reach 10 MPa to fabricate near-net shape composites. Pressure inside the nozzle could not conquer the surface tension to press the matrix into the voids of the fiber bundle if utilizing fused-extrusion process to fabricate CFRMMCs. In order to address this process limitation, wetting and wicking behaviors of molten metal on fiber bundles could be utilized. Hong [16] showed wicking behavior has been normally used to characterize the transferring ability of liquid to the inside of the fiber bundle. Nyoni [17] indicated the liquid could fill the voids inside the fiber bundle spontaneously and saturated without additional pressure by utilizing the wicking behavior. In order to improving the wetting performance, Singh [18] coated the metal on the surface of fibers to form appropriate interfaces. In addition, Qi [19] studied the effect of fiber orientation on mechanical properties of CF/Al composite, and found that total fiber bearing capacity in the tension direction was one of the significant factors in the UTS of the composites.

The low melting point alloys have the advantages of low melting temperatures and low surface tensions, especially the Pb50Sn50 alloy, the surface tension of which is 455 mN/m [20]. Because of the favorable wetting performance on Cu at low temperatures [21], Pb50Sn50 alloy is a suitable material for 3D printing process. In addition, the gamma rays shielding the performance of Pb50Sn50 alloy [22,23] mean that the material could be used in a high radiation environment, and the toxicity of lead element reduces with tin [22]. The material has potential application in the nuclear and aerospace fields.

Thus, a 3D printing method for continuous carbon fiber reinforced Pb50Sn50 low melting point alloy matrix composite is proposed and investigated in this paper. The mechanical performances of printed composite specimens were measured. The microstructures of fracture surface and interface were observed. Then, the wicking behavior between fiber and matrix in various temperatures was studied. The relationships between interfacial reactions and mechanical performance were discussed in detail. Finally, several composite parts were fabricated to validate the process for the manufacturing of complex structures. The method, which could be extended to other materials, obtained the favorable wetting and wicking performances between fiber and matrix.

## 2. Materials and Methods

### 2.1. 3D Printing Setup

**Figure 1:**
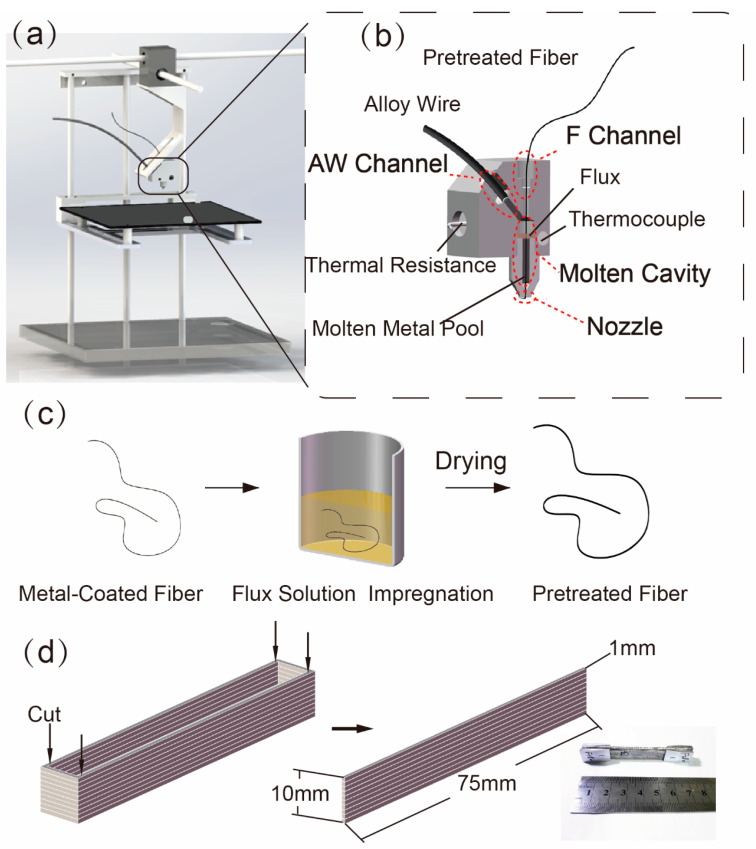
Schematic illustration of the continuous fiber reinforced metal matrix composites (CFRMMCs) fabrication process. (**a**) Structure of 3D printer of CFRMMCs; (**b**) sectional view of the printing head; (**c**) process of fiber pretreatment; (**d**) design of tensile specimen and the printed specimen.

As shown in Figure 1a, the structure of a CFRMMCs 3D printer was proposed. The printer comprised of the x-, y- and z- axis motion devices, the printing head and the substrate. Alloy wire and pretreated continuous carbon fiber were used as raw materials, and fed into the printing head. The designed printing head, which was composed of fiber channel (F channel), alloy wire channel (AW channel), molten cavity and nozzle, was shown in Figure 1b. The alloy wire was heated by thermal resistance (Figure 1b) and melted inside the AW channel, and then flowed into the molten cavity to form a molten metal pool. The temperature of printing was controlled by thermocouple (Figure 1b). The flux and pretreated fiber were sent into the molten cavity from the F channel. Then the wetting and wicking behaviors between molten metal and fibers occurred inside molten metal pool, and the voids inside fiber bundle were spontaneously filled. After that, composite monofilament was pulled out of the nozzle and accumulated to the desired structures. Therefore, composites, which were prepared by this process, could show suitable fiber-matrix interface and compact internal structure. Without additional pressure, the fiber and matrix formed the composite monofilament rapidly inside the printing head by wetting and wicking behavior.

### 2.2. Materials and Fiber Pretreatment

Alloy wire of Pb50Sn50 and Cu coated continuous carbon fiber were used as raw materials. The Cu coating could improve the wetting performance, because of the reaction wetting behavior between Cu and Sn. Alloy wires with a diameter of 2mm and a nominal composition of Pb50Sn50 (wt.%) were purchased from Shenzhen Xinghongtai Solder Co., Ltd (Shenzhen, China). The Cu coated continuous carbon fiber bundles (Cu-CF) were purchased from Suzhou Institute of Nano-Tech and Nano-Bionics, Suzhou, China. The standard properties of Cu-CF andPb50Sn50 alloywere listed in Table 1. There was a Ni coating between carbon fiber and the Cu coating, which would improve the bonding performance between copper and carbon. The flux of Pb50Sn50 alloy was purchased from Shenzhen Welsolo Co., Ltd (Shenzhen, China). The methylene dichloride (AR 99.5%) was obtained from Sinopharm Chemical Reagent Co., Ltd. (Xi’an, China).

Flux, which was used in the field of welding, could play a role in eliminating oxide layers and preventing the forming of new oxide layers. In previous experiments, excessive flux would prevent the matrix from being fed into the nozzle and flow out. Different weights of flux were solubilized in methylene dichloride to form 2%, 4%, 6%, 8%, 10% and 12% flux solution in a 250-mL beaker. The resulting solution was subsequently stirred until a homogeneous suspension was obtained. When the concentrations were 10% and 12%, the printing would keep stable and continuing. However, when the concentration was 12%, the extra flux would flow out of the nozzle. Hence, the concentration of 10% was chosen. The experimental details were shown in Supplementary Materials and Figure S1. The Cu-CF were immersed in flux solution for 1 min, and then dried at room temperature (25 °C), as shown in Figure 1c.

### 2.3. Sample Preparation

This process was performed on a 3D printer (COMBOT-I from Shaanxi Fibertech Technology Development Co., Ltd., Xi’an, China). The Pb50Sn50 and flux pretreated Cu coated carbon fiber (flux- Cu-CF) were used as materials, and the 10% concentration of flux solution was chosen. The process parameters were optimized, and the ultimate printing speed increased with the improving of printing temperature. The composites were designed as shown in Figure 1d. In order to test the tensile properties, the printed composites were cut into specimens with 75 mm × 1 mm × 10 mm in length, width and height, respectively, according to ASTM D3552-96(07) [24]. There were 5 samples per tensile test. The printing parameters were listed in Table 2. Wire feed ratio was the feeding distance of wire divided by actual printing distance.

### 2.4. Characterizations

Tensile properties were evaluated with an extension rate of 2mm/min. The microstructures of the composites were studied by scanning electron microscope (SEM, SU3500-Hitachi, Tokyo, Japan). The Back Scattered Electron (BSE) characterization was done using a BSE unit attached to SU3500 SEM. The Energy Dispersive Spectroscopy (EDS) characterization was also obtained by using an EDS camera attached to SU3500 SEM. The phases in the printed composite sample were evaluated using X-ray diffraction (XRD, Bruker D8 ADVANCE, Karlsruhe, Germany) with the 2θ range of 20° to 80°. The porosities of composites were measured by analyzing SEM images using the software of Image J. The thickness of the interfacial zone was measured by analyzing the SEM image and EDS map scanning image, using the software of Image J.

## 3. Results and Discussions

### 3.1. Mechanical Properties and Microstructure

#### 3.1.1. Influence of Process Parameters on Tensile Strength

**Figure 2:**
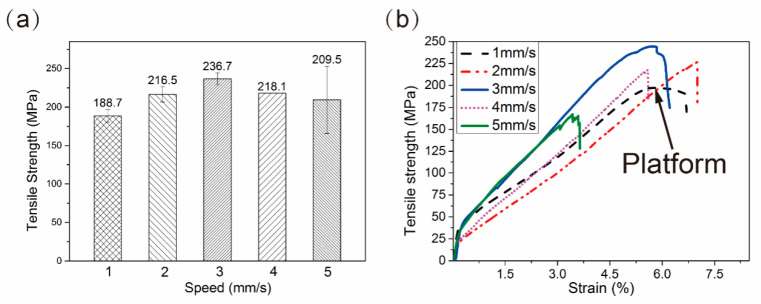
(**a**) Tensile strengths of CF/PbSn composites specimen S1, S2, S3, S4 and S5; (**b**) Typical strength is seen in strain curve of the printed CF/PbSn composites.

In order to study the variations of tensile performance with different process parameters, the composite specimens were printed at different printing speeds and temperatures. The tensile properties of specimens printed at 1 mm/s, 2 mm/s, 3 mm/s, 4 mm/s and 5 mm/s (S1 to S5) were shown in Figure 2a. With the improving of printing speed, the tensile strength increased first and then decreased, and reached the maximum value of 236.7 MPa, which was 7.1 times to that of Pb50Sn50 alloy (33.3 MPa) [25]. The typical tensile strength is seen in strain curves shown in Figure 2b. It was worth noting that a platform was formed on the curve of S1 around the value of ultimate tensile strength, and the fracture showed toughness. With the improvement of speed, the fracture mode transformed to brittle fracture. Additionally, it was obvious that several steps were on the curve of S5. The steps were formed by separation of layers during the test [25,26]. The bonding performance between two layers was weak at high printing speeds.

**Figure 3:**
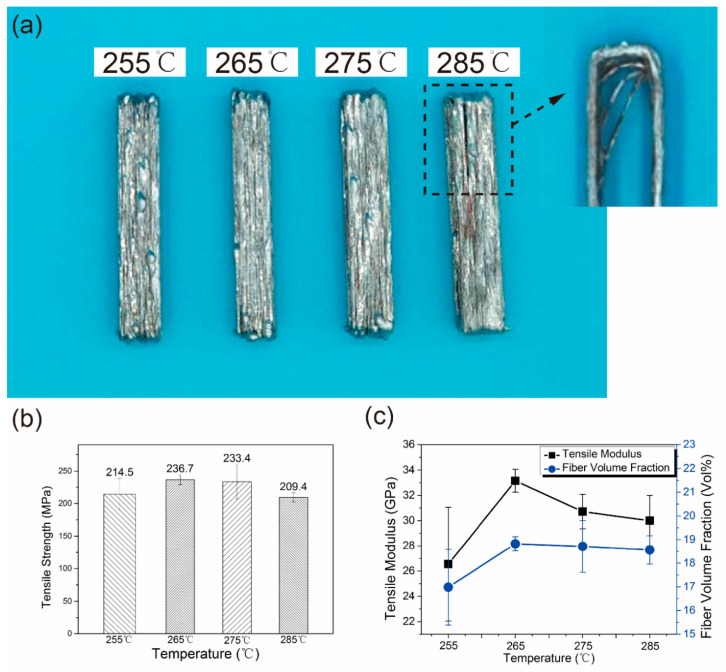
(**a**) Images of the composite structures printed at 255 °C, 265 °C, 275 °C and 285 °C observed with optical camera; (**b**) Ultimate tensile strength of the composite specimens S255, S265, S275 and S285. (**c**) Tensile modulus and fiber volume fraction of the composite specimens S255, S265, S275 and S285.

The printing temperature also had a great influence on tensile properties of composites. According to the previous exploration, the low printing temperature and high printing temperature would lead to poor interlayer bonding performance and the unevenly outflow of matrix, respectively. In this paper, the morphologies of the composite specimens printed at 255 °C, 265 °C, 275 °C and 285 °C (S255 to S285) were studied. The fluidity of molten metal was poor at 255 °C, and the spreading of molten metal on the surface of fiber was inhomogeneous. As shown in Figure 3a, small spheres could be observed on the surface of S255. The poor fluidity of matrix increased the pressure inside the nozzle and pressed the matrix outside to form spheres. With increasing temperature, the spheres disappeared gradually. However, when the temperature was high, the secondary heating effect, which would soften the printed composite around the nozzle, was improved. It was obvious that the fiber would deviate when printing the corner and the macro defects were formed, as shown in Figure 3a.It was worth to noting thatthe surface tension and solidification rate variation right before the solidus induced the surface roughness. Hence, the control of cooling rate was critical. According to the phase diagram, the solidus and liquidus temperatures of Pb50Sn50 were 183 °C and 228 °C, respectively, and the solidus temperature of formed CuSn was 227 °C. Hence, rapid cooling of the matrix to the temperature below 228 °C would stablise the shape of monofilament. When the printing temperature was 265 °C, the fluidity of matrix inside the nozzle was proper, and the monofilament would reduce to 228 °C rapidly. The printing quality of S265 was favorable. The control of the cooling rate must be matched with the alloy composition, and the further investigation was need to improve the process.

The tensile strengths were shown in Figure 3b. The tensile strengths of S265 and S275 were similar, and the value of S285 was poor owing to the apparent defects. In addition, the tensile modulus reached the maximum, which was 33.15 GPa, when the temperature was 265 °C (Figure 3c). The fiber volume fractions of the specimens were similar, except that of S255.The extra flowed spheres in S255 increased the volume of matrix, and the fiber volume fractions reduced.

#### 3.1.2. Fracture and Interfacial Microstructures

**Figure 4:**
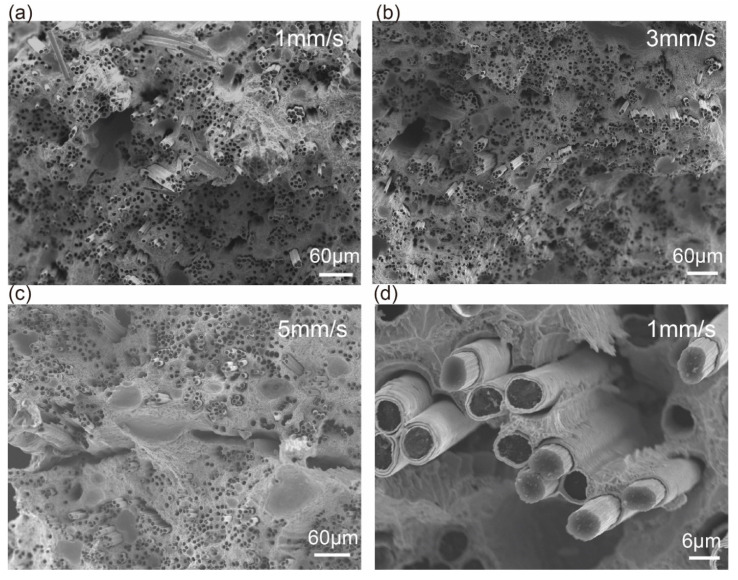
Morphologies of fracture surface for composite specimens (**a**) S1, (**b**) S3, and (**c**) S5; (**d**) Image of S1 specimen observed with higher magnification SEM.

Figure 4 shows the morphologies of fracture surfaces of S1, S3 and S5. The fracture surface of S1 was irregular, and the fibers pull-out could be observed. As can be seen from Figure 4b, the average lengths of fibers pull-out of S1 and S3 were 88.93 μm and 28.39 μm, respectively. When the speed was 5 mm/s, the fracture surface was flat and the macro defects were obvious, which induced the stress concentration. It is worth noting that the cracks occurred between the matrix and Cu coating during the tension procedure, as shown in Figure 4d. The morphologies of the fracture surfaces for composites printed at different temperatures were similar to that of S3 (shown in Figure S2).

**Figure 5:**
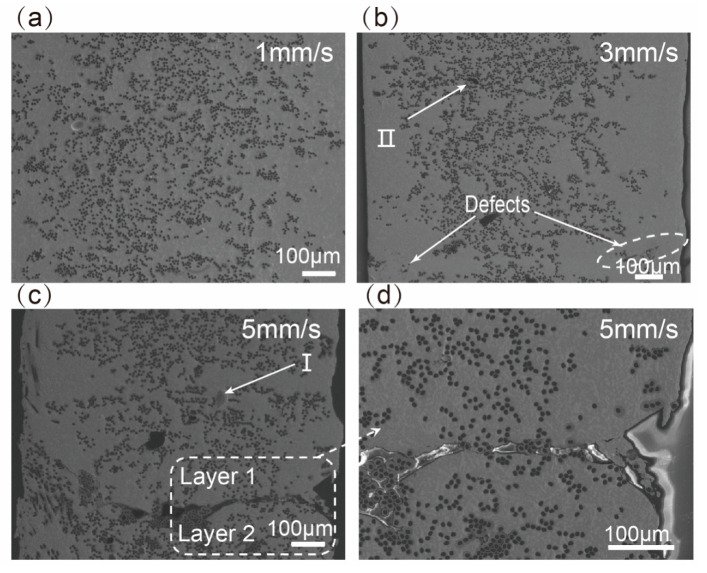
Images of interlayer morphology of the composite specimens (**a**) S1, (**b**) S3 and (**c**) S5 observed with back scattered electron (BSE); (**d**) Image of defect area observed in S5 with higher magnification BSE.

The microstructures of interfaces, which were composed of interlayer interface and fiber-matrix interface, were observed in this section. The forming of interlayer interface would be impacted by the heat from the nozzle, and the equation was shown below:

(1) $$Q=P\frac{L}{v}=\lambda S_{n}\Delta T\frac{L}{lv}$$

where *Q* was the heat, *P* was the heating power of nozzle, *L* was the total printing distance and *v* was the printing speed, λ was the thermal conductivity of nozzle, *S_n_* was the area of conduction heating surface, ΔT was the temperature difference, *l* was the heating distance. *Q* could be calculated by Equation (1) (the data of parameters were shown in Table 3), and the results were shown in Table 4. As shown in Figure 5, the bonding performance of S5 was poor. The upper layer could not totally bond with the lower layer in S5. When the printing speed decreased to 3mm/s, most of the defects disappeared (Figure 5b). By further decreasing the printing speed to 1mm/s, no crack was visible (Figure 5a) probably due to remelting of the printed surface at low speed. With the decreasing of *Q*, the porosities of composite increased sharply as shown in Table 4. In addition, there were two kinds of voids in composites, as shown in Figure 5b,c. One kind of voids was gas porosity (I). The undischarged air and volatilization of flux were left in the matrix, and formed the voids when the matrix solidified. The other kind of voids was fibers porosity (Ⅱ). The incomplete infiltration of the matrix led to forming of these voids around fibers. It was obvious that the voids inside S1 and S3 were significantly less than that inside S5, because the time of the printing process was plentiful to discharge the air and infiltrate the fiber when S1 and S3 were printed. It was worth noting that low porosities did not reduce the tensile properties of composites, such as S3, but the high porosities did. It was consistent with the results above.

**Figure 6:**
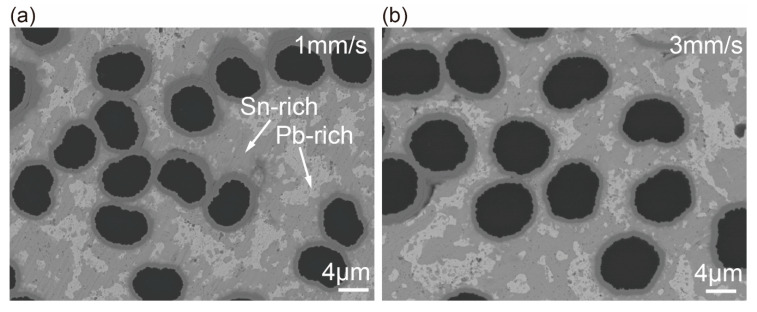
Image of fiber-matrix interface of the composite specimens (**a**) S1 and (**b**) S3 with higher magnification BSE.

The fiber-matrix interface was studied by BSE and EDS analysis. The morphologies of the fiber-matrix interface were shown in Figure 6. The matrix was wrapped on the fibrous surface, and no defect could be observed on the interface. EDS analysis indicated the dark-grey phase was Sn-rich and the light-grey phase was Pb-rich. The Sn-rich phases located around the profiles of individual fibers owing to the favorable wetting behavior between tin and copper. It was worth noting that the interfacial zone of S1 was obviously thicker than that of S3 (as shown in Table 5). The accumulation of interfacial reaction products increased the thickness, which is discussed in details later.

### 3.2. Discussions

#### 3.2.1. Wicking Behavior on Fiber Bundles

**Figure 7:**
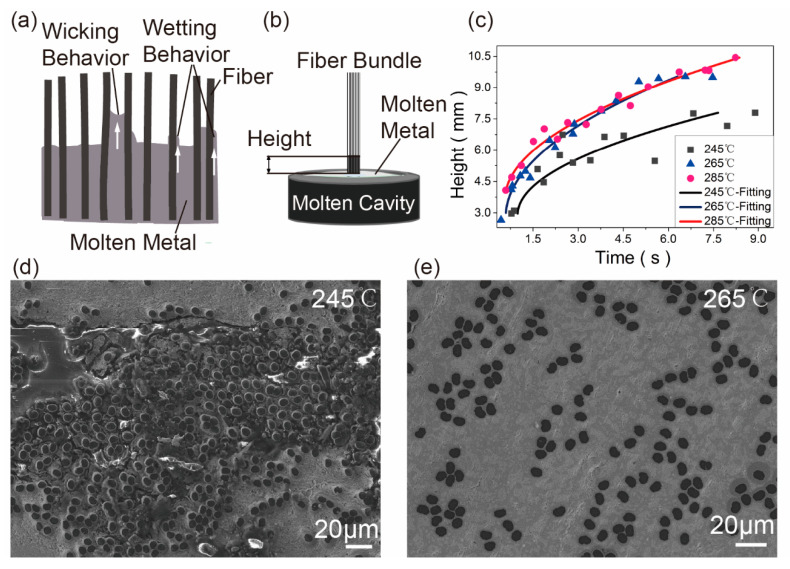
Wicking performance tests of CF/Pb50Sn50. (**a**) Mechanism of wicking and wetting behavior; (**b**) Schematic illustration; (**c**) Wicking performance of fiber bundles at 245 °C, 265 °C and 285 °C; Internal structure of composites printed at (**d**) 245 °C and (**e**) 265 °C.

During the printing process, the molten matrix would wet the profile of fibers by wetting behavior, and infiltrate into the fiber bundle and move along the fiber direction by wicking behavior [16] (Figure 7a). The degree of infiltration had a great influence on fibers porosity. Wicking behavior could be described as the model of the liquid rising in a bundle of capillary tubes. Pucci [27] and Washburn [28] showed the equation below:

(2) $$h^{2}\left(t\right)=\left[\frac{\left(c\bar{r}\right)}{2}\right]\frac{\gamma_{l}cos\theta_{\alpha }}{\varepsilon }t$$

where *h(t)* was the distance of the liquid front movement, *c* was a constant of the arrangement of equivalent capillary tubes, $\bar{r}$ was the mean capillary radius, $\gamma_{l}$ was the surface tension of liquid alloy, *θ_α_* was the dynamic advancing contact angle, *ε* was the liquid viscosity, *t* was the time of flow. The *h(t)* was increasing with the improving of wicking performance. According to Butler’s equation [29], $\gamma_{l}$ could be expressed using the density and surface tension of Sn and thermodynamic data as below:

(3) γl=γSn+RTSSnlnxSnSxSnb+1SSn(GSnSE−GSnbE)

where $\gamma_{Sn}$ was the surface tension of pure Sn, and $x_{Sn}^{S}$ and $x_{Sn}^{b}$ were the mole fraction in the surface and the bulk phase, respectively. *R* was the gas constant; *T* was the temperature in Kelvin, $S_{Sn}$ was the molar surface area of pure Sn, GSnSE and GSnbE were the partial excess Gibbs free energies of Sn in the surface and bulk phase, respectively. Using the thermodynamic data from the references [29,30], $\gamma_{l}$ could be calculated. By calculating, $\gamma_{l}$ reduced linearly with the temperature decreasing [30]. Therefore, the influence of printing temperatures was necessary to be studied.

The schematic of wicking performance test was shown in Figure 7b. The fiber bundle with flux was inserted into the molten matrix and the height of matrix rising was measured. The temperatures of molten matrix were 245 °C, 265 °C and 285 °C, respectively. It was obvious that the ultimate height was greatest at the temperature of 285 °C as shown in Figure 7c. With the decreasing of the temperature, the ultimate height reduced. According to Equation (2), *h* would improve with the increasing of temperatures, and *h*^2^ would increase linearly with t. The experimental results were consistent with the theoretical results. It was worth noting that the height would keep constant after rising for a moment. That might be attributed to the temperature differences above the molten cavity. When the metal was cool down and solidified, the height would keep constant. The Figure 7d was the SEM of the composite printed at 245 °C. The profile of fiber bundle was covered by Pb50Sn50 alloy by wetting behavior. However, the porosity was incompletely filled and only the ambient porosity was infiltrated. The wicking behavior could not drive the molten metal to infiltrate the fibers at 245 °C. The internal structure of composites printed at 265 °C was impact (Figure 7e). The wicking performance improved with the increasing of temperature until an impact internal structure was obtained. That was why the tensile strength of printed composites firstly increased and then kept constant with the increasing of printing temperatures. It was worth noting that the distribution of fibers was different in Figure 7d,e. This might be attributed the pressure formed by infiltration.

#### 3.2.2. Interfacial Reaction in 3D Printing Process

**Figure 8:**
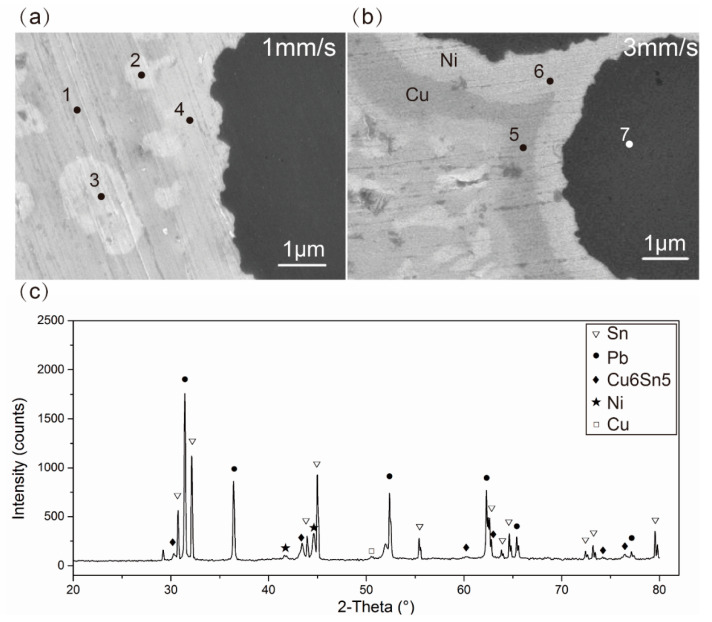
Images of fiber-matrix interface of (**a**) S1 and (**b**) S3 observed with higher magnification scanning electron microscope SEM; (**c**) x-ray diffraction (XRD) analysis of the interfacial reaction products.

It is well known that the degree of interfacial reactions was determined by the reaction time and temperature. In this process, the influence of reaction time was much greater than that of the reaction temperature, as shown in Table 5. This might be attributed to the narrow windows of printable temperature, and widely alterable reaction time. In order to study the influence of the reaction time, which was determined by the printing speed, the interfacial microstructural morphologies of the composites S1 and S3were observed, as shown in Figure 8.The EDS results (Table 6) showed the Cu coating on the surface of fiber was completely exhausted by interfacial reaction in S1.The Cu coating still existed in S3.The accumulation of formed intermetallic compound (IMC) on the surface of fiber would increase the thickness of the interface. It was worth noting that the IMC diffused into the matrix, owing to the shearing behavior between fiber and matrix during the printing process. The interfacial zone of S1 was thicker than that of S3. It might be attributed to the slower printing speed, which increased the accumulation time and decreased the diffusion rate. In addition, the interfacial reaction products were studied by XRD and EDS analysis. The EDS and XRD results (Figure 8c) showed the IMC was Cu6Sn5. Meanwhile, the harmful interfacial reaction product of Cn3Sn [31], which was produced in traditional manufacturing methods, was not observed in this process owing to the short process time. The element C in Microarea 3 and 4 may be induced by the organic impurities adsorbed on the sample surface.

The interfacial reaction had a direct influence on the mechanical performance of the composite. When the printing speed exceeded 3mm/s, the performance of interfacial bonding decreased and mechanical property reduced. This was because of the lack of interfacial reaction. The excessively interfacial reaction would also reduce the mechanical property. According to the EDS map scanning images, the thickness of Cu6Sn5 in S1 was 1.2μm, and the thickness of Cu6Sn5+residual Cu in S3 was 0.607μm (shown in Table 7). The contents of Cu6Sn5 or Cu6Sn5+residual Cuwere64.96% and 45.75% in the interfacial layer of S1 and S3, respectively. More Cu6Sn5 accumulated on the fibrous surface would reduce the tensile strength of fibers, owing to the poor tensile strength of Cu6Sn5 comparing with Cu. The thicker interfacial zone decreased the mechanical properties. This was why the tensile strength of S3, in which interface was thinner, reached the maximum. In addition, the platform on the tensile strength-strain curve of S1 showed toughness. On the one hand, the interface of S1 was rough, and the rough interface hindered crack propagation. On the other hand, the crack deflected at the interface, owing to the higher modulus of Cu6Sn5.

#### 3.2.3. Demonstration of 3D Printed CF/Pb50Sn50 Parts

**Figure 9:**
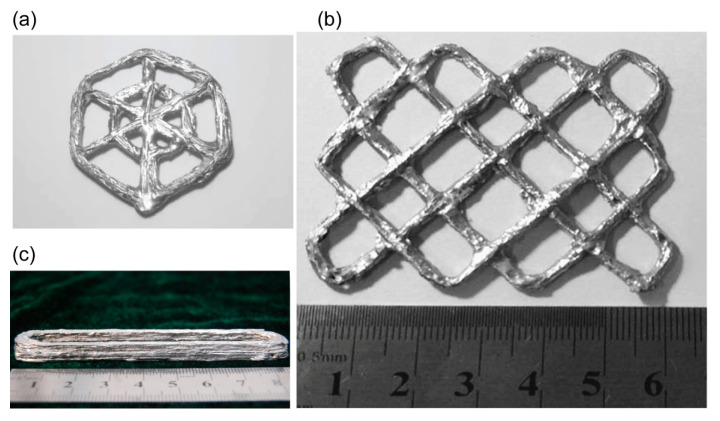
(**a**) Concentric hexagonal mesh grid; (**b**) Cross mesh grid; (**c**) Thin-wall part.

In order to demonstrate the unique advantages of this process in manufacturing of complex structures which were difficult to fabricate by traditional method, several kinds of complex parts were printed (Figure 9). As shown in Figure 9b, a grid with a length of 60mm, a width of 45mm and a thickness of 1.5mm was printed at the speed of 3mm/s, the temperature of 275 °C and the height of 0.3mm per layer. The continuous fiber distributed along the ribs, and the orientation of individual fiber bundle was independent. Although there were some deviations in the corners and cross sections, which need to be further optimized, this process had unique advantages in manufacturing complex structures of metal matrix composites in low cost.

## 4. Conclusions

A novel 3D printing process of CFRMMCs was proposed by using modified continuous carbon fiber as reinforcement phase and Pb50Sn50 alloy as matrix. The method solved the difficulties of the rapid combination of fiber and matrix, which resulted from the high surface tension and easy oxidation of molten metal, and improved the development of fabrication complex structural CFRMMCs. The mechanical properties of the composite specimens were studied, and the ultimate tensile strength reached 236.7MPa, which was 7.1 times that of Pb50Sn50 alloy. The apparent defects and voids in the composite could be eliminated by optimizing the printing temperatures and speeds. The wicking behavior between fiber and matrix would firstly increase and then keep constant with the improving of printing temperature. The interfacial reaction product was Cu6Sn5 and the harmful Cu3Sn was not observed. When the printing speed did not exceed 3mm/s, the thickness and roughness of fiber-matrix interface increased with the decreasing of printing speed. Hence, the specimens with the thicker interfacial zone (S1) showed lower tensile strength but toughness fracture. In addition, the complex structure composite parts could be printed by this process. Thus, the method had the advantages of low cost, high performance, short fabrication period and flexibility in fabrication of complex structures, ensuring the potential future applications in the aerospace, electronics and nuclear fields.

## Figures and Tables

**Table 1:** Properties of materials.

	Coating Thickness μm	Quantity of Individual Fibers	Tensile Strength MPa	Tensile Modulus GPa
Cu-CF	2.0(Cu)+1.0(Ni)	1K	2900	230
Pb50Sn50	/	/	33.3	8

**Table 2:** Printing parameters.

Group	Layer Height mm	Printing Temperature °C	Printing Speed mm/s	Wire Feed Ratio %
1	0.3	265	1, 2, 3, 4, 5	11
2	0.3	255, 265, 275, 285	3	11

**Table 3:** Data of parameters in Equation (1).

*λ* W/m∙°C	Δ*T* °C	*L* mm	*l* mm	*S_n_* mm^2^	*v* mm/s
401	240	2325	45	1	1, 3, 5

**Table 4:** Porosities in composites and calculated *Q*.

Printing Speed mm/s	Porosities		*Q* kJ
	Gas Porosities	Fiber Porosities	
1	1.64%	/	4.97
3	1.22%	2.84%	1.66
5	2.56%	11.92%	0.99

**Table 5:** Thickness of interfacial zone.

Printing Temperature °C	Printing Speed mm/s	Original Coating Thickness μm	Interfacial Layer Thickness μm	Standard Deviation μm
255	3	3	1.345	0.170
265	3	3	1.327	0.225
275	3	3	1.326	0.148
285	3	3	1.333	0.133
265	1	3	1.848	0.630

**Table 6:** Chemical composition of microareas 1–5 indicated in Figure 8a,b.

Number	Compositions (at %)					Possible Phases
	Sn	Pb	Cu	Ni	C	
Microarea 1	95.73				4.27	Sn-rich
Microarea 2	50.77	3.68	45.56			Cu6Sn5
Microarea 3		46.21			53.79	Pb-rich
Microarea 4	25.18		19.14	31.11	24.58	Cu6Sn5, Ni
Microarea 5			100			Cu
Microarea 6			2.45	97.55		Ni
Microarea 7	2.05		11.74		86.21	C

**Table 7:** Thickness of Cu+Cu6Sn5 vs. tensile strength.

Printing Speed mm/s	Cu+Cu6Sn5 Thickness μm	Interfacial Thickness μm	Content %	Tensile Strength MPa
1	1.200	1.848	64.96	188.7
3	0.607	1.372	45.75	236.7

## Supplementary Materials

The following are available online at https://www.mdpi.com/1996-1944/13/16/3463/s1, Figure S1: Optimization of solution concentration, Figure S2: Morphologies of fracture surface for composite specimens at different temperatures.
