# Supplementary material for: 3D Printing of Continuous Fiber Reinforced Low Melting Point Alloy Matrix Composites: Mechanical Properties and Microstructures
Source: Materials (Basel). 2020 Aug 6;13(16):3463. doi: 10.3390/ma13163463 (PMC7475899; doi:10.3390/ma13163463)
Supplement: Supplementary file 1 [file materials-13-03463-s001.pdf]

*Supplementary Materials*

# 3D Printing of Continuous Fiber Reinforced Low Melting Point Alloy Matrix Composites: Mechanical Properties and Microstructures

**Xin Wang [1,2], Xiaoyong Tian[1,2,*], Lixian Yin[1,2] and Dichen Li [1,2]**

[1] State Key Laboratory of Manufacturing Systems Engineering, Xi'an Jiaotong University, Xi'an 710049, China; wx369806199@hotmail.com (X.W.); yinlixian@stu.xjtu.edu.cn (L.Y.); xjtudcli@sina.com (D.L.)

[2] Shaanxi Provincial Rapid Manufacturing Engineering Technology Research Center, Xi'an 710049, China

* Correspondence: leoxyt@mail.xjtu.edu.cn; Tel.: +86-029-82665065

Received: 12 June 2020; Accepted: 03 August 2020; Published: date

### *1.1. Optimization of solution concentration*

Flux, which was used in the field of welding, could play a role in eliminating oxide layers and preventing the forming of new oxide layers. In this paper, the flux was added into the printing head and floated on the molten metal. The layer of flux would isolate the molten matrix from air and remove the oxide layer on the surface of Cu-CF. Different weights of flux were added into the printing head, and the printable distances were measured. As shown in Figure S1b, with the increasing of the weight of flux, the printable distance increased linearly. When the weight was more than 30mg, the flux would flow out of the nozzle and influenced the quality of printed composites. That was because excessive flux would prevent the matrix from being fed into the nozzle.

In order to keep printing, the weight of flux should remain constant. In this paper, the Cu-CF was pretreated with flux solution, and the consumed flux inside the printing head would be supplemented. The concentrations of 0%, 2%, 4%, 6%, 8%, 10% and 12% were chosen, and the whole printing distance was 150mm. As shown in Figure S1c, when the concentrations were 10% and 12%, the printing would keep stable and continuing. In addition, the extra flux would flow out of the nozzle when the concentration was 12%. Therefore, the concentration of 10% was proper.

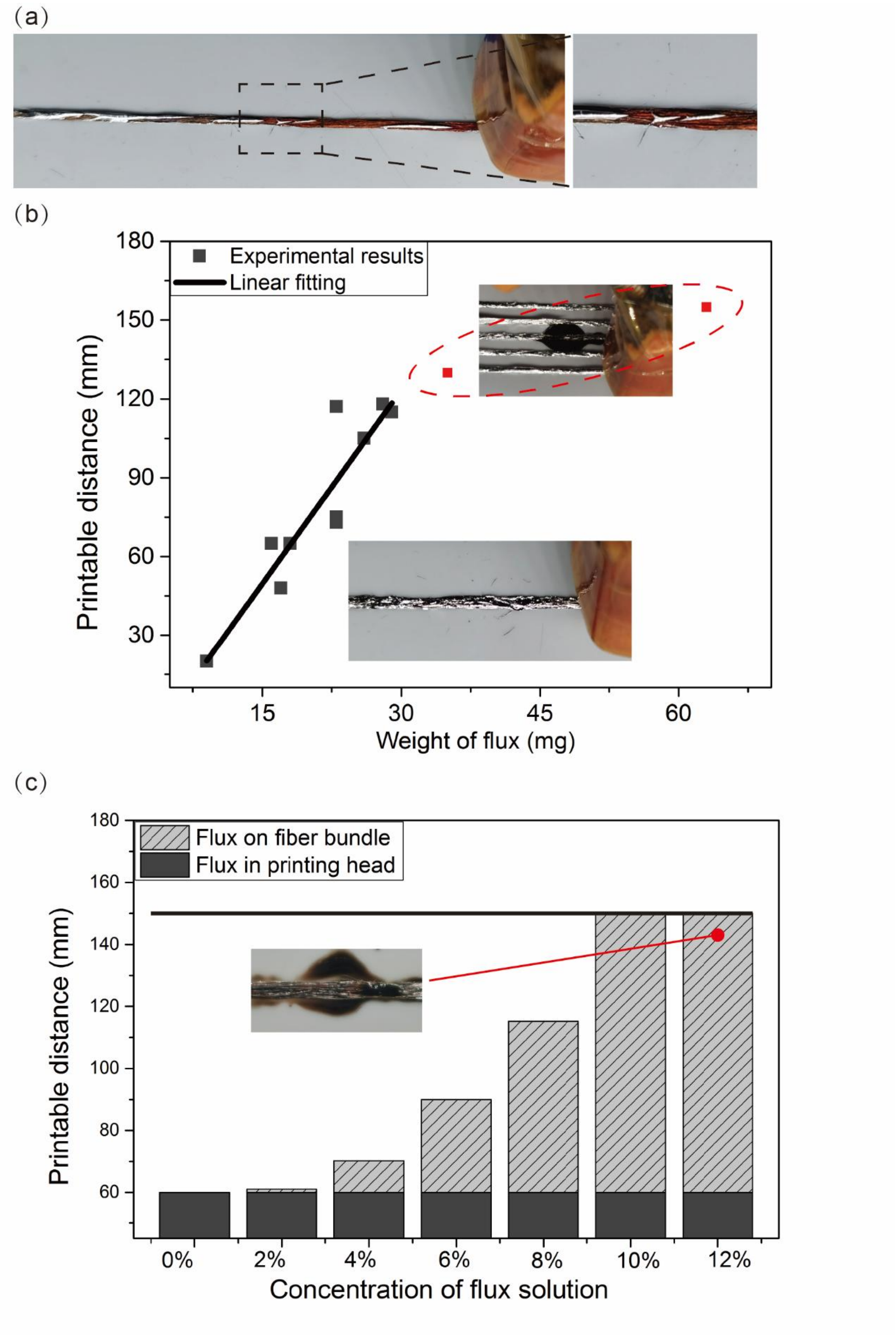


**Figure S1.** (a) The image of Cu-CF/Pb50Sn50 printed without flux; (b) The printable distances varied with the weights of flux inside the printing head; (c) The printable distances varied with the concentrations of flux solution.

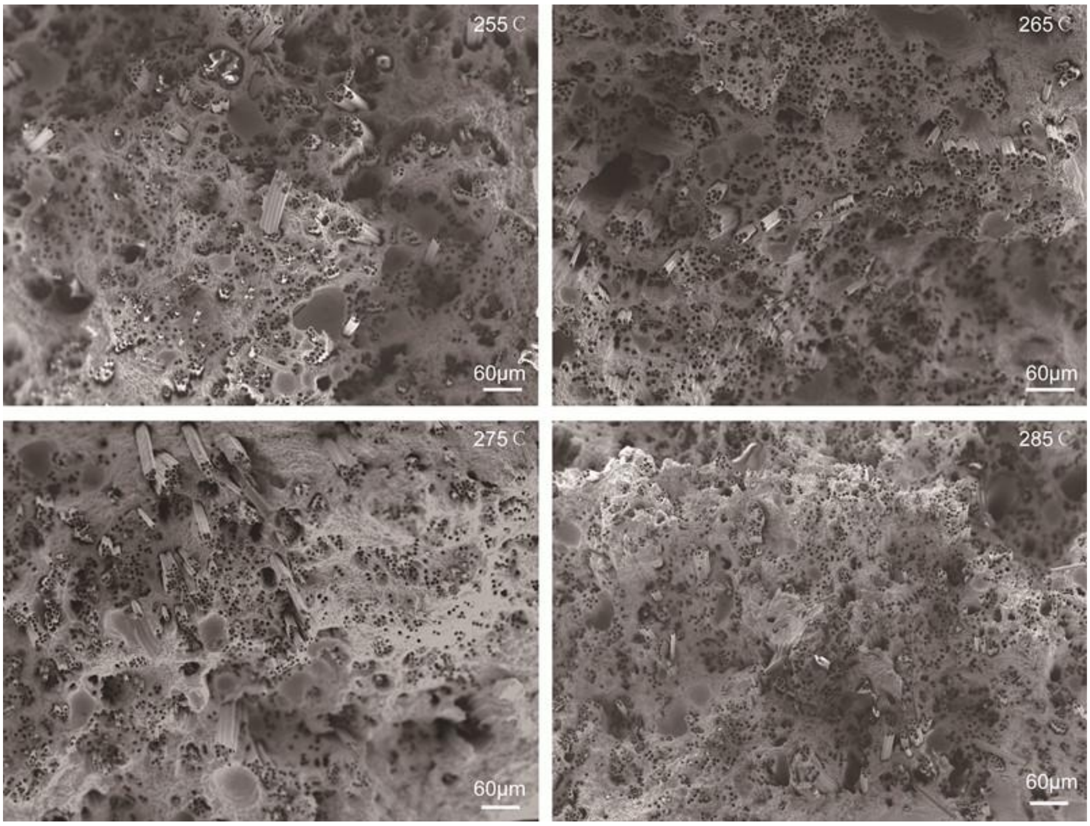


**Figure S2.** Morphologies of fracture surface for composite specimens at different temperatures.
